# Hemophagocytic Lymphohistiocytosis (HLH) in Patients with Tick-Borne Illness: A Scoping Review of 98 Cases

**DOI:** 10.3390/idr16020012

**Published:** 2024-02-21

**Authors:** Dorde Jevtic, Marilia Dagnon da Silva, Alberto Busmail Haylock, Charles W. Nordstrom, Stevan Oluic, Nikola Pantic, Milan Nikolajevic, Nikola Nikolajevic, Magdalena Kotseva, Igor Dumic

**Affiliations:** 1 Icahn School of Medicine at Mount Sinai, New York, NY 10029, USA; jevticd@nychhc.org (D.J.); alberto.busmail@gmail.com (A.B.H.); 2 Department of Medicine, NYC Health + Hospitals/Elmhurst, New York, NY 11373, USA; 3 Municipal University of São Caetano do Sul—USCS Bela Vista, Sao Paulo 01327-000, Brazil; mariliadagnon@gmail.com; 4 Department of Hospital Medicine, Mayo Clinic Health System, Eau Claire, WI 54703, USA; nordstrom.cw@mayo.edu; 5 Mayo Clinic College of Medicine and Science, Rochester, MN 55902, USA; 6 Department of Internal Medicine, Mayo Clinic Health System, Mankato, MN 56001, USA; oluic.stevan@mayo.edu; 7 Clinic of Hematology, University Clinical Center of Serbia, 11000 Belgrade, Serbia; drnikolapantic@gmail.com; 8 School of Medicine, University of Belgrade, 11000 Belgrade, Serbia; milannikolajevic10@gmail.com (M.N.); nikicke.nn@gmail.com (N.N.); 9 Internal Medicine Residency, Franciscan Health, Olympia Fields, Chicago, IL 60461, USA; magdalena.kotseva@franciscanalliance.org

**Keywords:** lymphohistiocytosis, hemophagocytic, tick-borne diseases, adult HLH, macrophage activation syndrome, *Ehrlichia* spp., *Anaplasma phagocytophilum*, *Rickettsia* spp., Powassan virus, Lyme disease, Hartland virus

## Abstract

Hemophagocytic lymphohistiocytosis (HLH) secondary to tick-borne infections is a rare but potentially life-threatening syndrome. We performed a scoping review according to PRISMA guidelines to systematically analyze the existing literature on the topic. A total of 98 patients were included, with a mean age of 43.7 years, of which 64% were men. Most cases, 31%, were reported from the USA. Immunosuppression was present in 21.4%, with the most common cause being previous solid organ transplantation. Constitutional symptoms were the most common, observed in 83.7% of the patients, while fever was reported in 70.4% of cases. Sepsis was present in 27.6%. The most common laboratory abnormalities in this cohort were thrombocytopenia in 81.6% of patients, while anemia, leukopenia, and leukocytosis were observed in 75.5%, 55.1%, and 10.2%, respectively. Liver enzyme elevation was noted in 63.3% of cases. The H-score was analyzed in 64 patients, with the mean value being 209, and bone marrow analysis was performed in 61.2% of patients. *Ehrlichia* spp. was the main isolated agent associated with HLH in 45.9%, followed by *Rickettsia* spp. in 14.3% and *Anaplasma phagocytophilum* in 12.2%. Notably, no patient with Powassan virus infection or Lyme borreliosis developed HLH. The most common complications were acute kidney injury (AKI) in 35.7% of patients, shock with multiple organ dysfunction in 22.5%, encephalopathy/seizure in 20.4%, respiratory failure in 16.3%, and cardiac complications in 7.1% of patients. Treatment included antibiotic therapy alone in 43.9%, while 5.1% of patients were treated with immunosuppressants alone. Treatment with both antibiotics and immunosuppressants was used in 51% of patients. Appropriate empiric antibiotics were used in 62.2%. In 43.9% of cases of HLH due to tick-borne disease, patients received only antimicrobial therapy, and 88.4% of those recovered completely without the need for immunosuppressive therapy. The mortality rate in our review was 16.3%, and patients who received inappropriate or delayed empiric therapy had a worse outcome. Hence, we suggest empiric antibiotic treatment in patients who are suspected of having HLH due to tick-borne disease or in whom diagnostic uncertainty persists due to diagnostic delay in order to minimize mortality.

## 1. Introduction

Hemophagocytic lymphohistiocytosis (HLH) is a rare but potentially fatal syndrome which was first described in 1939 as “histiocytic medullary reticulosis” [1]. HLH results from an overactivation of the immune system including CD8+ cytotoxic T cells, natural killer cells, and macrophages resulting in a systemic inflammatory process that is mediated by hemophagocytosis in bone marrow and extramedullary tissues. Several criteria have been developed to aid in diagnosis, including the HLH-94 and HLH-2004 [2,3]. The most widely used diagnostic criteria are the HLH-2004 criteria, which require five of the following eight features: cytopenias (two or more lineages of hemoglobin 9 g/dL or less, platelets < 100 × 10^9^/L, and/or neutrophils < 1 × 10^9^/L), fever ≥ 38.5 °C, hypertriglyceridemia and/or hypofibrinogenemia, elevated soluble CD25, splenomegaly, hemophagocytosis on biopsy, absent or low NK cell activity, and ferritin > 500 mcg/L [2]. Although HLH-2004 criteria were created for children and initially had not been validated for adults, the “HLH in adults“ Working Group of the Histiocyte Society recommended these diagnostic criteria in conjunction with clinical data [4]. After the publication of the recommendation, different groups showed the excellent discriminatory power of HLH-2004 criteria in identifying adult patients with HLH. Ferritin has a good negative predictive value, meaning that patients with normal ferritin levels are unlikely to have HLH. However, high ferritin is seen in various medical conditions and is non-specific, and one study demonstrated that only 19% of patients with HLH had ferritin levels above 50,000 mcg/L, and the values were higher in patients with hepatocellular and/or renal injury [5]. Soluble CD25 has the specificity of testing to 81% [6].

HLH is classified as a primary (familial) or a secondary disease. Primary causes include syndromes such as Chediak–Higashi and Griscelli syndrome, while secondary forms develop from infectious triggers, malignancy, autoimmune conditions, vaccines, or drug use [7,8,9,10,11]. In patients with rheumatic disease, HLH is commonly termed Macrophage Activation Syndrome (MAS) [12]. While viral infections are the most common trigger for HLH amongst adults, tick-borne infections such as *Anaplasma phagocytophilum*, *Ehrlichia chaffeensis*, and *Rickettsia rickettsii* have been previously reported as well. HLH secondary to tick-borne illness is a rare, yet life-threatening condition. The HLH syndrome overall mortality rate remains high at 41% [13]; however, there has been a significant improvement in the survival rate with the implementation of new treatment protocols over the last several years [12].

The clinical presentations, complications, and outcomes in patients suffering from secondary HLH in the setting of tick-borne infections are not well understood. Since both disease categories usually present with fever, variable degrees of cytopenia, and an increase in inflammatory markers, clinicians are often faced with challenging differential diagnoses. The appropriate recognition of HLH is of paramount importance, as delay in diagnosis directly corresponds to increased mortality [14].

Thus far, there have been no prospective studies or retrospective studies analyzing HLH in the context of tick-borne infections, and our understanding stems from individual case reports and case series. Hence, the objective of our scoping review is to systematically analyze the literature on this topic and describe the most common tick-borne pathogens associated with HLH and the clinical manifestations, complications, management, and outcomes among these patients.

## 2. Materials and Methods

We performed a scoping review of the literature following PRISMA guidelines by searching the PubMed/Medline database for articles reporting cases of tick-borne infections associated with HLH, using the following search terms: “tick borne illness and HLH OR tick borne illness and hemophagocytic lymphohistiocytosis OR anaplasmosis and HLH OR anaplasmosis and hemophagocytic lymphohistiocytosis OR babesiosis and HLH OR babesiosis and hemophagocytic lymphohistiocytosis OR borreliosis and HLH OR borreliosis and hemophagocytic lymphohistiocytosis OR ehrlichiosis and HLH OR ehrlichiosis and hemophagocytic lymphohistiocytosis OR Rickettsia and HLH OR Rickettsia and hemophagocytic lymphohistiocytosis OR Tularemia and HLH OR Tularemia and hemophagocytic lymphohistiocytosis OR Powassan virus and HLH OR Powassan virus and hemophagocytic lymphohistiocytosis OR Coxiella Burnetti and HLH OR Coxiella Burnetii and hemophagocytic lymphohistiocytosis OR Borrelia Burgdorferi and HLH OR Borrelia Burgdorferi and hemophagocytic lymphohistiocytosis OR lyme disease and HLH OR lyme disease and hemophagocytic lymphohistiocytosis OR tick borne illness and MAS OR tick borne illness and macrophage activation syndrome OR anaplasmosis and MAS OR anaplasmosis and macrophage activation syndrome OR babesiosis and MAS OR babesiosis and macrophage activation syndrome OR borreliosis and MAS OR borreliosis and macrophage activation syndrome OR ehrlichiosis and MAS OR ehrlichiosis and macrophage activation syndrome OR Rickettsia and MAS OR Rickettsia and macrophage activation syndrome OR Powassan virus and MAS OR Powassan virus and macrophage activation syndrome OR Coxiella Burnetti and MAS OR Coxiella Burnetii and macrophage activation syndrome OR Borrelia Burgdorferi and MAS OR Borrelia Burgdorferi and macrophage activation syndrome OR lyme disease and MAS OR lyme disease and macrophage activation syndrome”. All case reports and case series published from the date of the database inception to 10 July 2023 were analyzed.

**Figure 1:**
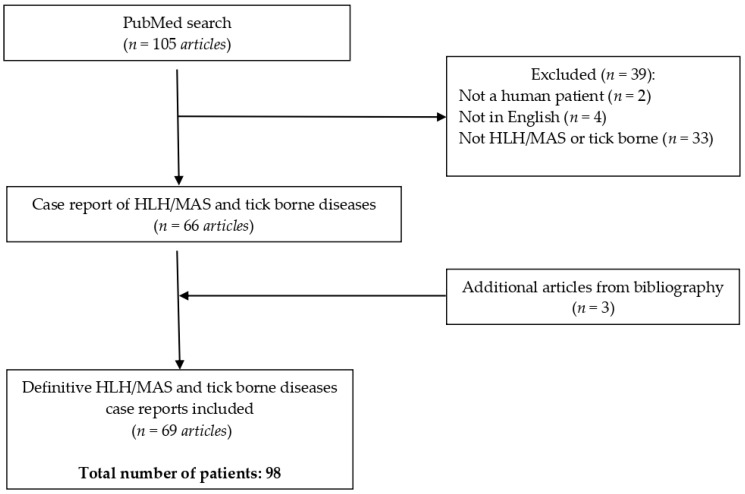
PRISMA table.

Two authors blindly and independently selected the cases (D.J. and S.O.). A senior author (I.D.) made a definitive choice when the two authors did not reach a consensus. All included cases met the criteria of having confirmed tick-borne disease and a high likelihood of HLH, as evidenced by the H-score and/or bone marrow (BM) analysis. The following were excluded from the analysis: articles published in languages other than English; case reports not providing an appropriate amount of information, or where the diagnosis was not certain; and those reporting non-human subjects. The final number of articles included was 69, which resulted in a total number of 98 patients (Figure 1) [15,16,17,18,19,20,21,22,23,24,25,26,27,28,29,30,31,32,33,34,35,36,37,38,39,40,41,42,43,44,45,46,47,48,49,50,51,52,53,54,55,56,57,58,59,60,61,62,63,64,65,66,67,68,69,70,71,72,73,74,75,76,77,78,79,80,81,82,83].

Immunosuppression was defined as the following conditions: malignancy on chemotherapy, solid organ transplant on immunosuppressants, autoimmune disease, end-stage renal disease (ESRD), splenectomy, and acquired immunodeficiency syndrome (AIDS). Constitutional symptoms were defined as chills, myalgia, asthenia, and fatigue. Sepsis was defined using SIRS criteria, with patients meeting at least two of four criteria, including a temperature >38 °C (100.4 °F) or <36 °C (96.8 °F), a heart rate >90 beats per minute, a respiratory rate >20 breaths per minute or PaCO_2_ < 32 mm Hg, a white blood cell count >12,000/mcL, and <4000 or >10% immature (band) forms.

In cases where the H-score was not reported, two independent researchers (D.J. and S.O) calculated the score if sufficient information was provided. In cases where there were not sufficient data for calculating the H-score, HLH was diagnosed based on the findings of the BM analysis.

## 3. Results

### 3.1. Demographic Characteristics

Our study included a total of 98 patients with a mean age of 43.7 years and a male predominance of 64.3%. When identifying the country of origin, 40.8% were unknown, 30.6% were from the U.S., 5.1% were from India, and the remaining 23.5% originated from 18 other countries.

The most common comorbidities in this patient cohort were hypertension in 11 patients (11.2%), chronic kidney disease (CKD) in 9 (9.2%), diabetes in 9 (9.2%), hypothyroidism in 7 (7.1%), and coronary artery disease (CAD) in 5 (5.1%). Immunosuppression was present in 21 (21.4%) patients, with the most common condition being solid organ transplantation and autoimmune disease history, documented in 8 patients in each group (8.2%).

### 3.2. Clinical Presentation

Constitutional symptoms were present in 82 (83.7%) cases, followed by fever in 69 (70.4%) cases. The criteria for sepsis were present in 27 (27.6%) patients. Laboratory evaluation was significant for thrombocytopenia in 80 (81.6%) patients, with the mean platelet count being 48.3. Anemia was present in 74 (75.5%), leukopenia was present in 54 (55.1%), and leukocytosis was present in 10 patients (10.2%). Liver function enzymes were elevated in 62 (63.3%) patients. The H-score was analyzed in 64 patients, with the mean value being 209 (range: 117–302).

The most commonly isolated tick-borne pathogen associated with HLH was *Ehrlichia* spp. in 45 patients (45.9%), followed by *Rickettsia* spp. in 14 (14.3%) and *Anaplasma phagocytophilum* in 12 (12.2%). The full list of pathogens is presented in Table 1. Seven patients (7.1%) were infected with two or more pathogens simultaneously.

### 3.3. Treatment and Outcome

The systemic complication of HLH associated with tick-borne diseases was most commonly acute kidney injury (AKI) in 35 (35.7%) patients, followed by shock with multiple organ dysfunction in 22 (22.5%), encephalopathy/seizure in 20 (20.4%), respiratory failure in 16 (16.3%), and cardiac dysfunction in the form of myocarditis, pericarditis, or cardiac arrest in 7 (7.1%) patients (Table 2).

Bone marrow analysis was conducted in 60 (61.2%) patients to establish HLH diagnosis.

The list of utilized treatment modalities is presented in Table 3. Treatment with both antibiotics and immunosuppressants was used in 51 (52%) patients, while antibiotics alone were used in 43 (43.9%) patients, and immunosuppressants alone were used in 4 (4.1%) patients.

Among immunosuppressants, steroids were utilized most commonly in 50 (51%) patients, and intravenous immunoglobulins were utilized in 10 (10.2%) patients. Other less commonly used medications included anakinra in five (5.1%) patients and cyclosporine in three (3.1%) patients. Etoposide, rituximab, and methotrexate were each used in two (2%) patients. Uncommonly used medication included colony-stimulating-factors, ribavirin, rifampin, ruxolitinib, eculizumab, and antimalarial agents in one patient each.

Antibiotics were usually started before immunosuppressants in 36 cases (36.7%), while immunosuppressants were started first in nine cases (9.2%), and both were started simultaneously in seven cases (7.1%). Appropriate empiric antibiotics were used in 61 patients (62.2%). The mean treatment duration with antibiotics was 13.8 days (range 1–90), and that of immunosuppressants was 33.1 days (range 5–61).

The overall mortality rate was 16.3%, and two patients (2%) were referred to hospice care. The characteristics of the patients who died are presented in Table 4. There were 11 male (68.85) and 5 female (31.2%) patients, with a mean age of 51.5 (range: 2–86) years. The highest mortality was observed in infections caused by *Ehrlichia* (*n* = 6, 37.5%), followed by *Rickettsia* spp. *Anaplasma phagocytophilum* (*n* = 3, 18.7% each), *Heartland virus* (*n* = 2, 12.5%), and, less commonly, *Bunyavirus* and *Orientia tsutsugamushi* (*n* = 1, 6.3% each). There was no significant underlying comorbidity noted in patients who died. Amongst the 16 patients who died, both antibiotics and immunosuppressants were used in 8 (50%), only antibiotics were used in 5 (31.2%), and only immunosuppressants were used in 3 (18.8%). Among the patients who died and received antibiotics (*n* = 13), appropriate and early empiric treatment was started in four (30.8%) patients, and inappropriate and delayed treatment was started in nine (69.2%) patients.

## 4. Discussion

### 4.1. Epidemiology

HLH is a rare and under-recognized disorder [7]. Previous studies demonstrated variable incidence depending on the population age and geographic location. A study in Japan demonstrated the incidence of 1 per 800,000 annual cases among both pediatric and adult patients [84]. One previous study reported an incidence of 1 in 100,000 among only pediatric patients under 18 years of age [85]. Prospective large-scale studies are lacking; hence, the global incidence has not been assessed to date.

Historically, HLH has been classified into two forms—familial and secondary [7]. Familial (or primary) HLH is caused by various inheritable genetic mutations, with the most common being the perforin-1 (PRF1) gene mutation, which encodes for perforin, a cytolytic protein of T cells and natural killer (NK) cells [7]. Primary HLH is more common in the pediatric population. Secondary HLH occurs due to a combination of immune predisposition and triggers, such as malignancy, rheumatic conditions, drugs, vaccines, and infections [7]. HLH associated with rheumatic diseases is also termed Macrophage Activation Syndrome (MAS) [86]. Infectious causes of secondary HLH are commonly viral—Epstein–Barr virus (EBV), human immunodeficiency virus (HIV), hepatitis B virus (HBV), hepatitis C virus (HCV)—or bacterial—*Mycobacterium tuberculosis* and *Pneumocystis jiroveci*. Viral HLH can be divided into non-EBV HLH, which is usually mild to moderate, and EBV-associated HLH, which is usually more severe and is the most common viral cause of HLH [87,88].

HLH in the setting of tick-borne infections might be particularly challenging since both conditions present with varying manifestations of cytopenias, fever, and constitutional symptoms. Our study demonstrates that secondary HLH triggered by tick-borne infections is most commonly reported in the USA. Males are almost twice as likely to be affected compared to females—64.3% and 35.7%, respectively. It is unclear whether this is related to genetic differences or the higher exposure of males to environmental risk factors, such as tick bites during hunting, gardening, and other outdoor activities. Tick-borne diseases do not seem to have gender predilection. Previous studies have demonstrated inconsistent results, with one study demonstrating that anaplasmosis is more common in males [89] while another reported a higher incidence of ehrlichiosis-associated HLH in females [90].

Patients with secondary HLH due to tick-borne infections had comorbidities similar to the rest of the general population. In this review, 21.4% of patients were immunocompromised. In these cases, it is difficult to determine whether the presentation of HLH was related to the infection itself or the state of immunosuppression. All of the immunocompromised patients were treated with antibiotics, except for one patient who received immunosuppressants and eventually expired [49]. Immunosuppression can also predispose to a relapse of tick-borne infections, as evidenced by a patient with multiple sclerosis on anti-CD20 therapy who presented with a relapse of babesiosis and HLH nine months after the initial treatment [83]. Both babesiosis and anaplasmosis are usually self-limited asymptomatic or minimally symptomatic infections in immunocompetent patients; however, in those with deficits in cellular or humoral immunity, these infections are often life-threatening and challenging to treat [89,91]. Hence, patients with secondary HLH due to anaplasmosis or babesiosis should have a thorough evaluation for immunodeficiency states.

### 4.2. Tick-Borne Pathogens Associated with Secondary HLH

The most common tick-borne pathogen associated with HLH is *Ehrlichia chaffeensis* in 45.9%. *Ehrlichia* spp. is transmitted by the Lone Star tick and is endemic to the Midwest, Southeast, and Southcentral regions of the US [92]. *Rickettsia* spp. and *Anaplasma* spp. were the second and third most common pathogens associated with HLH, respectively. Several types of Rickettsia have been described, including *Rickettsia typhi*, *tsutsugamushi*, *israelensis*, and *conorii*. A rare viral trigger of HLH that is transmitted by the Lone Star tick is Heartland virus, which was first isolated in 2009 in patients who presented with fever and cytopenias [93]. This virus is an emerging vector-borne illness and should be in the differential diagnosis, especially amongst patients who test negative for human monocytic ehrlichiosis (HME), anaplasmosis, and other pathogens transmitted through the *Ixodes* tick [94]. A lack of a response to appropriate empiric antibiotics, which typically cover bacterial tick-borne infections, should also raise suspicion for this culprit. One of the patients included in our review was discharged on antibiotic therapy, only to return to the hospital with worsening symptoms, and was found to be infected with *Heartland virus*. Ultimately the patient developed acute hypoxic respiratory failure and expired [43]. The delay in diagnosis might have, at least partially, contributed to the negative outcome. In our review, there were no cases of HLH associated with infections with *Powwasan virus* or *Borrelia* spp.

### 4.3. Clinical Presentation

During the development of HLH, activated CD8+ T cells produce a vast amount of interferon-gamma, leading to the overstimulation and expansion of both CD8+ T cells and macrophages. The activated CD8+ T and macrophages infiltrate and accumulate in the spleen, lymph nodes, liver, and other organs, resulting in an increase in cytokines that lead to end organ damage, which in turn results in the clinical symptoms manifested as SIRS or sepsis [95]. Complications of both the tick-borne infection and HLH overlap, and it is difficult to delineate whether manifestations are attributable to the infection or immune dysregulation in patients who suffer from these conditions. It is unclear why some individuals develop HLH in response to tick-borne infections while others do not, as well as why certain pathogens cause HLH at a higher rate (for example, *Ehrlichia* spp.) and some like Powassan virus and *Borrelia burgdorferi* have not been documented to trigger secondary HLH.

Cytopenias and liver dysfunction were frequently observed in laboratory investigations. Cytopenia occurs due to the process of hemophagocytosis in the bone marrow. Splenomegaly is frequently present in patients with HLH, while in tick-borne infections, it is usually a manifestation of babesiosis [96] and is not common in other tick-borne infections.

In our systematic review, 20.4% of patients had CNS manifestations on presentation or during the course of the disease. The dominant perspective is that HLH is triggered by an inflammatory cytokine storm by activated CD8+T and macrophages, which is believed to cause CNS involvement by crossing the blood–brain barrier [97]. The methods of pathogens entering the CNS are generally categorized based on the specific cellular route they utilize, including intercellular, transcellular, or leukocyte-facilitated transfer [98]. In patients with HLH triggered by *Anaplasma* spp., 28% had CNS manifestations such as an altered mental status, confusion, syncope, and dysarthria. Neurological symptoms associated with anaplasmosis are comparatively less frequent when compared to other tick-borne illnesses like ehrlichiosis or Lyme disease. Headache is a common manifestation of anaplasmosis, noted in up to 40% of individuals affected [89]. The involvement of the central nervous system in the form of meningitis and encephalitis is rare, with some experts suggesting that less than 1% of patients with anaplasmosis experience CNS manifestations [99]. CNS manifestations secondary to *Babesia* spp. are unusual, with two cases reported, and include confusion, slurred speech, and ataxia. Both cases had favorable outcomes and were empirically treated with broad-spectrum antibiotics [100]. Instances of unilateral facial, cerebral infarction, and visual impairment in *Rickettsia* spp. infections have been documented previously as well [101,102,103].

Gastrointestinal (GI) manifestations as an initial presentation were found in 30% of patients, which included: abdominal pain, diarrhea, and vomiting. Previous studies demonstrated that the GI symptoms are a common initial presentation in patients with anaplasmosis; hence, it is difficult to differentiate HLH secondary to tick-borne infections from isolated tick-borne infections based on GI symptomatology [89].

Kidneys are commonly involved with AKI, reported in 35.7% of patients. Two cases necessitated the initiation of renal replacement therapy; however, the injury was completely reversible in both [74]. This incidence is lower compared to the previous incidence of 69% of AKI in HLH [104] but higher compared to the incidence of AKI in some tick-borne infections—for example, anaplasmosis, where it is estimated to be around 15.5%. A previous study also demonstrated that patients with ehrlichiosis who required an ICU level of care more frequently presented with an AKI [105].

Uncommon complications reported in our review included skin necrosis, serous membrane inflammation (pleuritis/pericarditis), myocarditis, and intracranial hemorrhage [25,48,67,74,82]. A petechial rash associated with fever may be the earliest dermatologic signs of tick-borne disease [106]. This manifestation is not commonly seen in HLH; however, it may be a presenting symptom of HLH associated with tick-borne infections, which can aid in appropriate diagnosis. Interestingly, in one of the patients, a skin biopsy was conducted for diagnostic purposes in the presence of the rash, and it showed findings of leukocytoclastic vasculitis [67]. The pathogen isolated in this case was *Rickettsia conorrii*, which has previously been shown to be associated with fulminant purpura and small vessel vasculitis due to its tropism for endothelial cells [107]. Timely recognition and antibiotic treatment are paramount, as fulminant purpura can result in tissue necrosis, disseminated intravascular coagulation, and, often, fatal outcomes [107].

Our review includes five patients with myocarditis, and it is interesting that in four out of five cases, the isolated pathogen was *Ehrlichia* spp. [48,74]. The elevation of cardiac enzymes, hemodynamic instability, and the development of supraventricular and ventricular arrhythmias are presenting signs that clinicians should be aware of and which can uncommonly lead to cardiac arrest [48,49].

### 4.4. Diagnosis

The diagnosis of tick-borne infections depends on the causative agent and usually includes serologic testing, polymerase chain reaction, peripheral blood smear, and, rarely, tissue culture [108]. Bone marrow biopsy and analysis are commonly carried out to help establish the diagnosis of HLH. In our review, 61.2% of patients underwent BM analysis. Biopsies typically demonstrate the infiltration of tissues with lymphocytes, histiocytes, and hemophagocytosis [109]. Hemophagocytosis is a pathologic feature of HLH. However, Cetica et al. demonstrated that no more than 40% of patients had hemophagocytosis at the moment of diagnosis of HLH [110]. Additionally, diagnosis should not be delayed if hemophagocytosis is not found in the bone marrow sample. Since it is not exclusively specific for HLH, some authors consider it as one of the less important diagnostic criteria [111,112]. In summary, HLH is a syndrome where diagnosis should take into account the clinical presentation, significant laboratory markers, and pathology findings, avoiding overreliance on a single diagnostic modality.

### 4.5. Treatment

The mainstay of treatment for HLH is a combination of immunosuppressants and cytotoxic agents. Immunosuppressive choices are broad, with unknown benefits, and include corticosteroids, cyclosporine, etoposide, and others [103,113]. In our review, two patients received triple therapy consisting of corticosteroids, etoposide, and cyclosporine [30,46]. Intravenous immunoglobulins (anti-thymocyte globulin) and allogeneic hematopoietic cell transplantation have also been used in refractory HLH [109]. In our review, systemic corticosteroids were most commonly used in half of the patients. Other agents can be considered as well, and 31 (31.6%) patients were placed on more than one immunosuppressant.

For secondary HLH, targeted treatment for the trigger is of the utmost importance. In cases of tick-borne diseases, this treatment includes doxycycline for Lyme disease, HME, *Rickettsia* spp., and anaplasmosis [108]. For babesiosis, the treatment includes atovaquone and azithromycin. For viral pathogens, the therapy is supportive [114]. The role of immunosuppressants in secondary HLH has been questioned. Namely, in HLH-94, those patients were excluded, and there are not enough data regarding this clinical scenario. Although the timely introduction of triple therapy could be life-saving for patients with secondary HLH, sometimes, a dose modification and treatment duration may be required [115]. The addition of cyclosporine in the first line along with dexamethasone end etoposide did not improve outcomes significantly [113]. In 43.9% of cases of HLH due to tick-borne disease, patients received only antimicrobial therapy, and 88.4% of those recovered completely without the need for immunosuppressive therapy.

### 4.6. Outcome

The mortality of secondary HLH is not well established, and the majority of available information comes from clinical case reports [116]. The mortality rate in our review was 16.3%, which is significantly lower compared to the previously reported overall mortality of HLH at 41% [117] but higher than the mortality of tick-borne diseases, which in most severe cases, is up to 10% [108]. A prior study reported survival rates of 25.1% and 82.4% in patients with EBV-HLH and autoimmune disease-associated HLH, respectively [118]. EBV-related HLH mortality reaches 25–45%; however, it can reach up to 95% if not treated [87,88]. HIV-HLH mortality is around 31% [88]. Survival in patients with HLH and malignancy differs depending on the primary cancer and ranges from 18% to 55% [119]. Malignancy-related HLH is most commonly associated with hematologic malignancies and lymphomas. In this patient population, mortality can be up to 80%, although with novel treatments such as ruxolitinib, tocilizumab, and anakinra, mortality has been somewhat reduced. In patients with solid tumors, HLH is sometimes associated with chemotherapy used to treat the tumor and not the malignancy itself [120]. Overall, compared to other secondary causes of HLH, tick-borne-associated HLH has a higher survival rate.

The highest mortality was observed in infections caused by *Ehrlichia* spp. (38%), followed by *Rickettsia* spp. *Anaplasma phagocytophilum* (19% each), *Heartland virus* (13%), and, less commonly, *Bunyavirus* and *Orientia tsutsugamushi* (6% each). None of the patients infected with *Babesia* spp. died. Research suggests that mortality is high in HME due to a low level of NK cell activity and a lack of toll-like receptors (TLR) in *Ehrlichia* spp,. which is necessary for NK activity [17]. There was no significant difference in mortality in those infected with multiple pathogens; however, it is hard to draw conclusions in this setting due to the limited number of patients in our review. Patients who were adequately started on appropriate empiric antibiotics had an overall higher survival rate in our systematic review.

## 5. Conclusions

HLH secondary to tick-borne infections is uncommon, but the exact incidence is difficult to estimate due to the rarity of the disease and the lack of prospective studies. Men are more commonly affected than women, and most of the cases were reported from the USA. About 20% of all patients were immunocompromised, and *Ehrlichia* spp. is the most common tick-transmitted pathogen that is a culprit of HLH. Complications of the disease were various, with the most common ones being acute kidney injury, shock, respiratory failure, and seizure/encephalopathy, while cardiac complications such as myocarditis and pericarditis were seldom reported. The mortality in patients of HLH triggered by infection due to tick-borne disease in this review was 16%. Inappropriate empiric antibiotics and a delay in antibiotic administration were associated with higher mortality. Empiric and appropriate antibiotics should be considered early to decrease the mortality risk, as diagnostic testing is often delayed in patients with HLH secondary to tick-borne infections. The mortality of HLH secondary to tick-borne infections is lower compared to the previously reported mortality of isolated HLH yet higher than that of tick-borne infections. Future prospective studies are needed to assess the incidence of HLH in the setting of tick infection and better define therapeutic algorithms that can improve survival.

## 6. Limitations

This study has a few notable limitations. First, due to the nature of this type of review, some high-quality cases might have been omitted if they were not written in English or published in journals indexed in PubMed/Medline. Additionally, inherent to the nature of this review is publication bias. As features of sepsis defined by SIRS criteria and the presentation of HLH can overlap, this has likely led to a certain number of false positive presentations categorized to have sepsis. Finally, the sample size is relatively small.

## Figures and Tables

**Table 1:** Pathogens identified as potential causes of HLH.

Pathogen	Number of Patients (%)
*Ehrlichia* spp.	45 (45.9%)
*Rickettsia* spp.	14 (14.3%)
*Anaplasma* spp.	12 (12.2%)
*Babesia* spp.	10 (10.2%)
*Orientia* spp.	8 (8.2%)
*Coxiella* spp.	5 (5.1%)
*Heartland virus*	3 (3.1%)
*Bunyavirus* spp.	1 (1%)

**Table 2:** Systemic complications in patients with HLH and tick-borne infections.

Complications	Number of Patients (%)
Acute kidney injury	35 (35.7%)
Shock with multiple organ dysfunction	22 (22.5%)
Encephalopathy/seizure	20 (20.4%)
Respiratory failure	16 (16.3%)
Myocarditis/Pericarditis/Cardiac arrest	7 (7.1%)

**Table 3:** Overview of the treatments used in patients with HLH secondary to tick-borne infections.

Antibiotics (*n* = 94, 95.9%)		Immunosuppressants (*n* = 55, 56.1%) *			
Appropriate empiric (*n* = 61, 62.2%)	Not appropriate empiric/unknown (*n* = 33, 33.7%)	Steroids (*n* = 50, 51%)	IVIG (*n* = 10, 10.2%)	Anakinra (*n* = 5, 5.1%)	Cyclosporine (*n* = 3, 3.1%)

* A small subset of patients received other immunosuppressant treatments, including etoposide, rituximab, cyclosporine, methotrexate, and eculizumab. IVIG—Intravenous immunoglobulins.

**Table 4:** Characteristics of patients who died in our systematic review (references: [18,22,24,25,26,42,43,48,49,59,62,71,82]).

Publication	Age	Sex	Comorbidities	Pathogen	Immunosuppression	Antibiotics
Dahm CN, et al., 2020 [48]	68	M	NA	*Ehrlichia* spp.	Corticosteroids	Vancomycin and cefepime (admission). Doxycycline (5th hospital day)
Saha A, et al., 2022 [49]	70	M	ESRD, kidney transplant	*Ehrlichia* spp.	Corticosteroids and etoposide	Doxycycline and caspofungin (7th day after the initial symptoms)
Sharma S, et al., 2022 [42]	34	M	NA	*Rickettsia typhi*	Corticosteroids and etoposide	Doxycycline and Ciprofloxacin (1st hospital day)
Mitma AA, et al., 2021 [59]	72	M	Multiple myeloma	*Ehrlichia* spp.	Corticosteroids and tocilizumab	Vancomycin and cefepime (admission). Doxycycline was added afterwards.
Carlson AL, et al., 2018 [22]	*	M	DM, COPD, HTN, ischemic CMP, hypothyroidism, RA (prednisone, methotrexate, and adalimumab)	Heartland virus	Corticosteroids and etoposide	Vancomycin, meropenem, ampicillin, acyclovir, and doxycycline
Leal-López VF, et al., 2020 [18]	2	M	NA	*Rickettsia typhi*	Corticosteroids	Amoxicillin (symptoms onset). Doxycycline (third week)
Cabler SS, et al., 2020 [26]	7	F	NA	*Ehrlichia* spp.	Corticosteroids and etoposide	Doxycycline
Yi J, et al., 2017 [62]	32	M	NA	*Anaplasma phagocytophilum*	Corticosteroids, etoposide, and IVIg	/
Yi J, et al., 2017 [62]	53	M	NA	*Anaplasma phagocytophilum*	Corticosteroids, etoposide, and IVIg	/
Nakano A, et al., 2017 [71]	86	F	HTN	Bunyavirus	Corticosteroids	Not specified which, but prescribed on diagnosis
Saha A, et al., 2022 [49]	66	M	ESRD on HD, DM, kidney transplant	*Ehrlichia* spp.	Corticosteroids, tocilizumab, and IVIg	/
Lin YH, et al., 2014 [25]	34	F	Drug abuse, HCV infection	*Orientia tsutsugamushi*	Corticosteroids	Ceftriaxone and minocycline (1st hospital day).
Dahm CN, et al., 2020 [48]	60	F	NA	*Ehrlichia* spp.	/	Piperacillin-tazobactam and levofloxacin (1st hospital day). Doxycyclin (3rd hospital day)
Chandramohan D, et al., 2023 [82]	71	F	DM, HTN, dyslipidemia	*Rickettsia typhi*	/	Ceftriaxone
Liu S, et al., 2023 [43]	60	M	Asplenia, HTN, coronary arthery disease	Heartland virus	/	Doxycycline
Tsiodras S, et al., 2017 [24]	57	M		*Anaplasma phagocytophilum*		Meropenem and doxycycline (3rd hospital day)

NA—not applicable; DM—diabetes mellitus; M—male; F—female; COPD—chronic obstructive pulmonary disease; HTN—hypertension; CMP—cardiomiopathy; RA—rheumatoic arthritis; HCV—Hepatitis C infection; ESRD—end-stage renal disease; HD—hemodyalisis. *: unknown age.

## Data Availability

All data are publicly available.
